# Evaluation of the Effects of Different Types of Resin Cement Systems on the Push-Out Bond Strength of the Fiber Post to Intracanal Dentin in Anterior Primary Teeth

**DOI:** 10.1155/2023/8645083

**Published:** 2023-11-13

**Authors:** Ali Nozari, Boshra Rasoolzade, Zahra Jowkar, Seyed Ahmadreza Hamidi, Mohammad Jowkar

**Affiliations:** ^1^Department of Pediatric Dentistry, School of Dentistry, Shiraz University of Medical Sciences, Shiraz, Iran; ^2^Oral and Dental Disease Research Center, Department of Operative Dentistry, School of Dentistry, Shiraz University of Medical Sciences, Shiraz, Iran; ^3^Department of Oral and Maxillofacial Surgery, School of Dentistry, Shiraz University of Medical Sciences, Shiraz, Iran; ^4^Department of Prosthodontics, School of Dentistry, Tehran University of Medical Sciences, Tehran, Iran

## Abstract

**Background:**

This study aimed to evaluate the effects of using three different resin cements on push-out bond strength (PBS) of fiber posts to root canal dentin of primary teeth.

**Methods:**

Fifty primary canines were randomly divided into five experimental groups according to the type of the luting agent used for fiber post cementation after endodontic treatment and post space preparation as the following: Group 1: glass ionomer cement (GIC), Group 2: flowable resin composite, Group 3: etch and rinse (E&R) resin cement, Group 4: self-etch (SE) resin cement, Group 5: self-adhesive (SA) resin cement. The PBS values of the cemented fiber posts to root canals were measured. The data were statistically analyzed using a one-way analysis of variance, followed by Tukey's post hoc test.

**Results:**

The highest mean PBS value was observed in Group 4 (SE resin cement), followed by Group 3 (E&R resin cement), and the lowest PBS value belonged to Group 1 (GIC), followed by Group 5 (*p*-values < 0.05). The mean PBS of Group 3 (E&R resin cement) was significantly higher than those of Groups 1 (GIC), 2 (flowable resin composite), and 5 (SA resin cement) (*p*-values < 0.05). However, no significant difference was found between the mean PBS of GIC and SA resin cement (*p*-value >0/05).

**Conclusion:**

Using SE resin cement for fiber post cementation in primary anterior teeth showed the best results, followed by E&R resin cement. The lowest PBS was observed for GIC, followed by SA resin cement.

## 1. Introduction

Maintaining the healthy condition of primary dentition impacts children's well-being [1]. The most prevalent chronic disease of childhood is still dental caries, despite the recent decrease in the rate of dental caries in children [2]. Dental caries or physical trauma in the primary dentition can lead to the partial or complete loss of coronal tooth structure [3, 4].

Restoration of severely damaged primary anterior teeth is challenging for pediatric dentists [5]. The lost tooth structure should be restored to prevent possible problems associated with early tooth loss, such as esthetic problems, loss of vertical dimension, reduced masticatory efficiency, speech problems, nutritional problems, development of parafunctional habits such as tongue thrusting, and functional problems such as malocclusion and space loss [6]. Moreover, early tooth loss may lead to psychological burdens [7]. Therefore, attempts should be made to preserve the primary dentition's integrity until the primary teeth exfoliate normally.

After endodontic treatment in severely damaged anterior primary teeth, intracanal posts may be necessary before restoring teeth to increase the resistance of the restoration to masticatory forces and mechanical loads and restore the morphology of the crown [8]. Moreover, intracanal posts are used in teeth with severe structure loss to improve and ensure the retention of the restoration to the tooth [8]. Various posts are available for use in pediatric restorative dentistry, such as short posts with resin composite, polyethylene ribbon posts, metallic posts with macro-retention, prefabricated carbon fiber and glass fiber posts, and premanufactured orthodontic wire in different forms [5, 9, 10]. Prefabricated fiber-reinforced resin composite posts possess desirable features such as mechanical properties similar to dentin, biocompatibility, and high fatigue and corrosion resistance [5].

The post's systems intracanal retention is an essential factor in the longevity of resin composite restorations in severely damaged pulpotomized primary teeth [11]. Post length is a determining factor in post retention in root canal space [12]. Post length in permanent teeth has been suggested to be at least equal to the crown height or two-thirds of the root length for providing resistance to occlusal forces and better stress distribution [12]. However, for restoration of primary teeth, only a short length of each selected post system should be used to prevent interference of the post material with the subsequent smooth transition to permanent teeth or physiological root resorption of primary teeth [13]. On the other hand, the fiber posts are placed passively in the root canals; therefore, choosing the appropriate luting material for fiber post cementation is very important in the post's retention and the restoration's final success [8, 14, 15].

Various materials have been previously used for fiber post cementation in primary teeth [5, 16–18]. A common luting material for fiber post cementation in primary teeth is flowable resin composite [16]. Glass ionomer cement (GIC) has also been previously used for post cementation in primary teeth [18]. Different resin cements have been frequently used in the permanent teeth for fiber post cementation [19]. However, there is a lack of evidence in the literature concerning using different types of resin cements, including etch and rinse (E&R), self-etch (SE), and self-adhesive (SA) resin cements for fiber post cementation in primary teeth. Therefore, this study aimed to evaluate the effects of using three different resin cements on the push-out bond strength (PBS) of fiber posts to root canal dentin of primary teeth.

## 2. Materials and Methods

### 2.1. Tooth Preparation

The study protocol was approved by the Research and Ethics Committee of Shiraz University of Medical Sciences (Protocol #IR.SUMS.DENTAL.REC.1401.015).

Fifty caries-free human primary canines without root curvature, external root resorption, caries, cracks, and enamel defects were used in this study. Moreover, the collected teeth had sound roots with no more than one-third apical resorption. The reason for the extraction of the collected teeth was orthodontic treatment. The purpose of the research and applying the extracted teeth for this experimental study was explained to the patient's parents. A blinded, calibrated operator carried out all of the procedures of this experimental study. The collected teeth were stored in a 0.5% chloramine T solution at 4°C for a period no longer than 1 month before use after being cleaned with a periodontal curette.

Standard coronal access cavities were prepared after cutting the crowns of the teeth transversely 2 mm above the cementoenamel junction using a water-cooled low-speed cutting machine (Mecatome T201 A, Presi, Grenoble, France). The working length of each canal was considered the length of the initial file at the apical foramen minus 1 mm.

K-files (MANI, Utsunomiya Tochigi, Japan) were used to perform the complete pulpectomy to a size #45k file. The canals were irrigated entirely between files with 2 ml of normal saline. After completing filing, the canals were dried with paper points and obturated using the packing technique with a calcium hydroxide paste with iodoform (Metapex, Meta Dental, Korea) with the tip of a syringe. The obturated roots were kept in 100% humidity for 48 hr at 37°C.

To prepare the post space with a 3 mm length, approximately coronal 4 mm of paste in the obturated roots was removed with a round bur on a low-speed handpiece, and a base of GIC (GC Gold Label 1 Luting and Lining) with a thickness of 1 mm was placed over the Metapex.

### 2.2. Fiber Post Preparation

For preparing the posts, three mm of each glass fiber post (White post; FGM, Joinville, SC, Brazil) was sectioned using fissure bur on a high-speed handpiece with water spray. The length of the used fiber posts was considered as 3 mm, ensuring no interference with normal root resorption. Therefore, fiber post lengths were measured with a probe before post cementation. Moreover, post-fitment was checked in the canals. A fiber post with size 1 or 2 was selected for each root according to the dimension of the post relative to the root canal. After preparations of the fiber posts, the surfaces of the fiber posts were cleaned with 95% ethyl alcohol and air dried. Moreover, the post spaces were cleaned with distilled water and were gently air-dried with an air syringe and paper points.

### 2.3. Experimental Groups

The prepared roots were randomly assigned to five experimental groups (*n* = 10) according to the type of the luting agent used for fiber post cementation. The manufacturer and chemical composition of the materials utilized for cementing fiber posts in the current study are presented in Table 1.

The following methods were carried out for each group:

Group 1: GIC (GC Gold Label 1 Luting and Lining) was used for fiber post cementation. GIC was manipulated according to the manufacturer's recommendations and then used to cement fiber posts.

Group 2: The prepared post spaces were etched with 35% orthophosphoric acid (Scotchbond acid, 3M ESPE, St. Paul, MN, USA) for 15 s, then rinsed for 20 s and dried with paper points. An E&R adhesive bonding system (Adper Single Bond 2; 3M ESPE, St. Paul, MN, USA) was applied in two layers using microbrushes according to the manufacturer's recommendations. Moreover, the adhesive was also applied on the surface of the prepared fiber posts to strengthen their bonding to the utilized resin composites. Fiber posts were inserted in the prepared post spaces after a thin layer of a flowable resin composite (Filtek Ultimate Flowable, 3 M, USA) was applied in the canal.

Group 3: After etching the post space the same as Group 2, an E&R adhesive system (Excite DSC, Ivoclar-Vivadent, Schaan, Liechtenstein) was applied in two layers using microbrushes according to the manufacturer's recommendations. Afterward, the fiber posts were cemented with an etch-and-rinse resin cement (Variolink II, Ivoclar-Vivadent, Schaan, Liechtenstein). The base and catalyst of the resin cement were mixed in a 1 : 1 ratio, and the mixed resin cement was used for fiber post cementation according to the manufacturer's recommendations.

Group 4: An SE resin cement (ED Primer II/Panavia F2.0, Kuraray, Osaka, Japan) was used for fiber post cementation according to the manufacturer's instructions. After mixing an equal amount of liquid ED primers A and B, the prepared mixture was applied for 20 s on the post space walls and then gently air-dried for 5 s. An equal amount of pastes A and B were mixed and applied on the post space and the post surface for fiber post cementation.

Group 5: A SA resin cement (TheraCem, Bisco, Inc., Schaumburg, IL, USA) was used for fiber post cementation according to the manufacturer's instructions.

In each experimental group, the cement was applied on the post and into the post space using a lentulo spiral (Dentsply-Maillefer, Ballaigues, Switzerland). After placing the post into the canal with a slight vibratory motion, the post was firmly pressed for 5–10 s, and the excess cement was cleaned with a microbrush. Light polymerization of the resin cements and resin composite was performed using a light-curing unit (VIP Junior, Bisco, Schaumburg, IL, USA) at 800 mW/cm^2^ through the cervical portion of the root for 40 s at the buccal and lingual surfaces. A resin-modified GIC (GC Fuji II LC, GC Corporation Tokyo, Japan) was placed on the coronal part of the bonded roots to create a tight coronal seal. After completing the cementation procedures, the samples were stored for 24 hr in 100% humidity at 37°C.

### 2.4. PBS and Failure Mode Analysis

**Figure 1:**
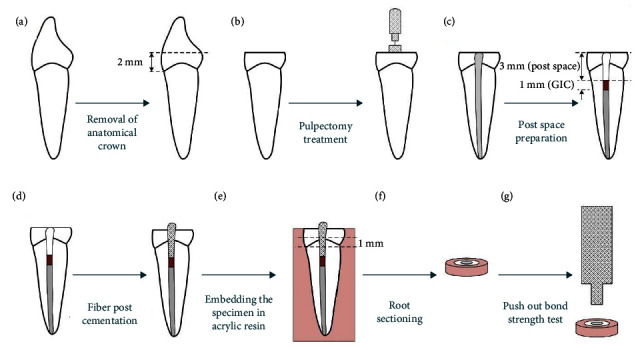
Schematic representation of the specimen preparation procedures: (a) removal of the anatomical crowns 2 mm above the cementoenamel junction; (b) pulpectomy treatment; (c) post space preparation (GIC, glass ionomer cement); (d) fiber post cementation; (e) embedding the specimen in acrylic resin; (f) root sectioning in the middle of the prepared area to obtain a 1-mm thick slice; (g) push-out bond strength test.

After embedding the bonded roots in acrylic resin, the roots were sectioned perpendicular to their long axis using a slow-speed cutting machine (Mecatome T201 A, Presi, Grenoble, France) in the middle of the prepared area to obtain a 1-mm thick slice from this root region. The thickness of the root sections was verified using a digital caliper (Digimess Direct, Sao Paulo, Brazil). The PBS test was performed using a universal testing machine (Instron Z020, Zwick Roell, Ulm, Germany) at a load of 5 kN. The crosshead speed of the applied compressive load was 0.5 mm/min on the center of the post in an apico-coronal direction until the shear stresses along the bonded interface dislodged the post. The specimen preparation procedures have been schematically illustrated in Figure 1.

For calculating the PBS in megapascals (MPa), the recorded load at failure in kilograms-force (kgf) for each specimen was divided by the interfacial area (*A*) of the post fragment, which corresponds to the bonded area in square millimeters (mm^2^). The bonded area was calculated as the lateral surface of a truncated cone using the following formula: *A* = *π* (*R* + *r*) (*h*^2^ + (*R* − *r*)^2^)^0.5^, where *π* is approximately 3.14, *R* and *r* represent the coronal and apical post radii, respectively, and h is the section height. However, since the PBS values were obtained in kgf, they need to be transformed to MPa using the formula MPa = kgf × 9.8/area.

The failure modes were determined under a stereomicroscope (Carl Zeiss, Oberkochen, Germany) at 40×magnification for debonded specimens and categorized as follows: (a) Cohesive failure in dentin, (b) Cohesive failure in post or cement, (c) Adhesive failure between dentin and cement, (d) Adhesive failure between post and cement, (e) Mixed failures consisting of a combination of two or more failure modes [19].

### 2.5. Statistical Analysis

The normality of the data was checked using the Shapiro–Wilk test. The data were statistically analyzed using a one-way analysis of variance (ANOVA) to evaluate the effect of the luting agent on the PBS of the fiber posts to root canal dentin. The PBS values of different groups were compared using Tukey's post hoc test. All the analyses were done using SPSS software version 17 (SPSS Inc., Chicago, USA). *p*-Values less than 0.05 were considered statistically significant.

## 3. Results

**Figure 2:**
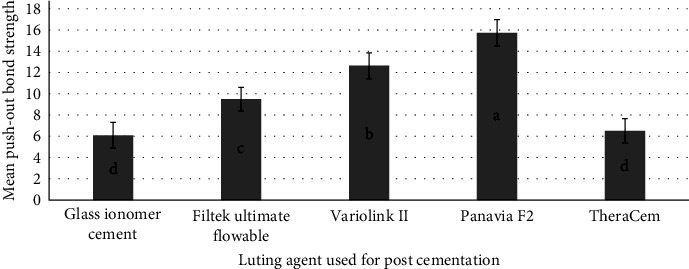
Mean push-out bond strength (± SD) in MPa of the experimental groups. Mean values represented with different superscript letters in the column are statistically significantly different (*p* < 0.05).

The mean PBS values (± standard deviation; SD) in MPa are presented in Figure 2. The one-way ANOVA revealed significant differences among all study groups (*p*=0.001). Post hoc analysis with Tukey's test was performed for pair-wise comparisons. The mean PBS of Group 4 (Panavia F2) was statistically significantly higher than other experimental Groups (*p*-values < 0.05). The mean PBS of Group 3 (Variolink II) was statistically significantly higher than that of the groups 1 (GIC), 2 (Filtek Ultimate Flowable), and 5 (TheraCem) (*p*-values < 0.05). Mean PBSs of Groups 5 (TheraCem) and 1 (GIC) were statistically significantly lower than other experimental groups (*p*-values < 0.05). However, no significant difference between PBS of Groups 1 and 5 was found (*p*-value > 0/05).

**Figure 3:**
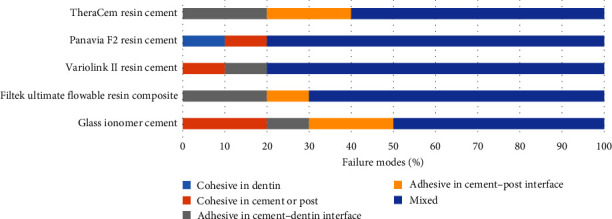
Distribution of failure mode frequency.

The distribution of failure mode frequency of the experimental groups is presented in Figure 3. The predominant failure mode was the mixed failure in all study groups.

## 4. Discussion

This study evaluated the effects of using three different resin cements on the PBS of fiber posts to root canal dentin of primary teeth. According to the results of the present study, the highest PBS was observed for the SE resin cement (Pavania F2), followed by the E&R resin cement (Variolink II). Moreover, GIC, followed by the SA resin cement (TheraCem), showed the lowest PBS value.

Following endodontic treatment in anterior teeth with excessive crown destruction, root dentin is the only available tooth structure for crown reconstruction. Therefore, a post should be used to retain the coronal restoration [20]. However, when restoring severely destroyed anterior primary teeth, intracanal retention should not interfere with normal exfoliation of the primary teeth [8]. Therefore, the standard prepared post space should be at most 3 mm in length inside the canal [8].

A prefabricated fiber-reinforced resin composite post is one of the most commonly used posts for restoring severely damaged anterior primary teeth [21]. Previously, fiber posts were used successfully in severely damaged primary teeth in experimental studies [16, 20]. A clinical study also showed that fiber post presented an acceptable clinical performance concerning material fracture and retention when restoring severely decayed primary anterior teeth at the 12-month follow-up period [11]. Moreover, enhanced fracture resistance of the teeth restored with fiber post and resin composite build-up was observed previously compared to no post [22]. Fiber posts also demonstrated a reduced risk of root fracture due to their similar modulus of elasticity to root dentin [23].

Therefore, prefabricated fiber-reinforced resin composite posts are appropriate treatment options for restoring severely damaged anterior primary teeth. Thus, in the present study, prefabricated fiber posts were used to assess the influence of different luting agents on the PBS of the posts to root canal dentin.

Besides post selection, luting cement is also an influential factor in post retention and its bond strength to root canal dentin [24].

One of the luting agents used previously for fiber post cementation is GIC [25]. The primary mechanism of adherence of GIC to the dentin substrate is a chemical bond between the carboxylate groups formed during the acid–base setting reaction of the GIC and the calcium ion of the hydroxyapatite (HAP) [26]. No dentin conditioning is needed before post cementation with GIC [27]. GIC demonstrated similar PBS to an SA resin cement when GIC was used for fiber post cementation in a previous study [25]. Therefore, GIC was used as a luting agent for fiber post cementation in the current study.

Flowable resin composite is commonly used as a luting agent for fiber post cementation in primary teeth. Flowable resin composites have low viscosity and thus can present good adaptation to root canal walls [28]. Moreover, flowable resin composites can perform as flexible intermediate layers and help relieve stresses during polymerization shrinkage of resin composite restoration due to their low modulus of elasticity and low viscosity [28]. Therefore, a flowable resin composite was another luting agent used for fiber post cementation in this study.

Recently, different types of resin cements have been used for fiber post cementation in permanent teeth due to their advantages, such as high PBS to root canal dentin, excellent mechanical properties, and stability after thermocycling evaluation [29]. Compared to resin composite for post cementation, dual-cured resin cements demonstrate increased working time, reduced chairside time, great mechanical properties, a high degree of conversion, enhanced polymerization, and excellent bond strength [22, 30]. Three main types of resin cements include E&R, SE, and SA resin cements [19, 29]. E&R resin cements need acid etching and an adhesive bonding agent before applying the resin cement [19]. SE resin cements only require the application of an SE primer or an SE adhesive bonding agent [19]. Recent advances in developing resin cements with fewer procedural steps and shorter working times led to the introduction of SA resin cements [25]. However, no previous study compared the effects of different types of resin cements on PBS of fiber posts to root canal dentin of primary teeth. Therefore, the main purpose of this study was to explore the effect of the type of resin cement on the bond strength of fiber posts to root canal dentin. Variolink II, Pavavia F2, and TheraCem were used in the present study as E&R, SE, and SA resin cements, respectively.

Panavia was used as an SE resin cement in the present study. Panavia F2 resin cement is applied following the application of a SE adhesive primer [19]. In addition to the micromechanical bonding, Panavia F2 can create a stable chemical bond with calcium of HAP of enamel and dentin due to the presence of 10-methacryloyloxydecyl dihydrogen phosphate (MDP) monomer in its formulation [31]. Calcium phosphate bonds resulting from the interaction of functional monomers of Panavia F2 with HAPs can remain stable for long periods under hydrophilic conditions [31]. In the present study, Panavia F2 showed the highest PBS value, which may be justified by the enhanced hybridization resulting from the chemical interaction between 10-MDP and calcium of HAP. Another explanation for the higher PBS of the SE resin cement used in the current study compared to the E&R resin cement and flowable resin composite is the simultaneous demineralization and resin penetration and, thus, equal depth of dentin demineralization and monomer penetration in the SE adhesive systems [32].

According to the results of the present study, the E&R resin cement and flowable resin composite showed significantly lower bond strength than the SE resin cement. This finding could be justified by applying acid etching before the E&R resin cement and flowable resin composite. The density and diameter of dentinal tubules are higher in primary teeth than the corresponding values in the permanent teeth. This leads to a lower amount of intertubular dentin and, therefore, less available substrate for bonding to the adhesive in primary teeth [32, 33]. On the other hand, in primary teeth, compared to permanent teeth, there is a thicker amount of peritubular dentin, which is demineralized faster in the etching process [32]. Therefore, deeper penetration of the acidic conditioner occurs into the dentinal tubules in primary teeth compared to the permanent teeth, which leads to stronger demineralization. Considering the differences in acid penetration into the dentinal tubules of primary and permanent teeth, a weaker acidic solution (with a mild pH level) or shorter conditioning time is recommended for primary teeth [34]. In the present study, the acid etching was applied for 15 s according to the manufacturer's recommendations, which might result in deep demineralization in the root canal dentin of the primary teeth. This deep demineralization can lead to the discrepancy between the degree of dentin demineralization after etching and the penetration of the adhesive bonding agent, which was applied following acid etching [34]. Therefore, the lower PBS of the E&R resin cement and flowable resin composite compared to that of the SE resin cement in the present study can be justified by the probable presence of deep layers of demineralized dentin that was not completely saturated with the adhesive bonding agent. This unsaturated etched dentin can act as a mechanically weak area and decrease bond strength and nanoleakage [34]. Thus, to prevent excessive demineralization in the etching process, it would be beneficial to assess the effect of shorter conditioning time and lower concentrations of acid etching before the application of the E&R resin cement on the PBS of the fiber posts to root canal dentin of primary teeth in future studies.

Reducing the application steps in pediatric dentistry is preferable because using multistep agents in children is time-consuming and difficult [35]. Therefore, SA resin cements, which are one-step systems, simplify and shorten the process of fiber post cementation and can be especially beneficial for use in uncooperative children [25, 35]. TheraCem was used as a SA resin cement in this study. The results of this study demonstrated that the SA resin cement had lower PBS compared to other types of resin cements and flowable resin composites. The SA resin cements do not need dentin surface pretreatment before applying the resin cements [29]. The smear layer is not removed and remains unmodified before applying the resin cement [29]. Therefore, the presence of the unmodified smear layer can interfere with the penetration, hybridization, and bonding of the SA resin cement with dentin. These explanations can justify the lower PBS of the SA resin cements compared to other types of resin cements and flowable resin composite in the current study.

Another finding of the present study was that fiber posts cemented with SE and E&R resin cements demonstrated higher PBS values than those cemented with flowable resin composite. This finding can be attributed to the dual cure polymerization mechanism of the used SE and E&R resin cements, which enhances the polymerization of the cement in the apical area compared to the flowable resin composite [30].

In the present study, GIC showed a lower PBS value than the flowable resin composite, E&R resin cement, and SE resin cement. However, the present study found no significant difference between PBS of GIC and the SA resin cement. Lower PBS of GIC compared to those of the other luting cements can be attributed to some drawbacks of GIC, such as sensitivity to dehydration, initial brittleness, risk of contamination during its self-cure setting reaction, and greater risk of void formation [36]. The chemical reactions between the HAP and the carboxyl groups in the chemically activated GIC are similar to the interactions between HAP and SA resin cements [25]. This explanation may justify the similar PBSs obtained for GIC and SA resin cement in the present study. The predominant adhesion mechanism of the groups cemented with E&R, or SE resin cement and flowable resin composite was micromechanical bonding at the adhesive interface following the application of the adhesive bonding agent or primer and formation of the hybrid layer [25, 37, 38]. However, micromechanical bonding is fainter in the case of GIC and SA resin cement [25]. The chemical bonding mechanism of the SA resin cement and GIC may positively affect the bond strength durability of fiber post to root canal dentin. Thus, comparing the effect of GIC or SA resin cement with other types of resin cements on the long-term bond strength of fiber posts to root dentin is recommended.

Another possible influential factor on the bond strength of the fiber posts to root canal dentin is the root canal filling materials during endodontic therapy [16]. It has been reported that the root canal filling materials containing eugenol, such as zinc oxide eugenol, can negatively affect the bond strength of the fiber posts cemented with resin cements to the root canal dentin [16]. However, root canal filling materials containing calcium hydroxide, such as Metapex, showed higher post bond strength to the root canal's dentin surfaces than zinc oxide [16]. Metapex is easily resorbed from the periapical area and showed higher post bonding strength to the dentin surfaces of the root canal compared to zinc oxide [16]. Therefore, Metapex was used as the root canal filling material in the present study.

Different tests have been previously used to assess the retention of posts in the root canal space, such as conventional shear, microtensile pull-out, and push-out tests [24]. The fractures occur parallel to the dentin cement or post cement interface in PBS tests, leading to a more favorable condition within a clinical setting [24]. Therefore, it has been reported that better results have been obtained by a push-out test compared to a conventional shear test when measuring the bond strength of fiber posts to root canal dentin [24]. Moreover, the micro-PBS test presents fewer pretest failures, less variability in mechanical testing results, lower standard deviation, and more homogenous stress distribution than the microtensile bond strength test [39]. Therefore, PBS was used to assess the effects of different luting agents on the bond strength of fiber posts to root dentin. Moreover, canal diameters were measured carefully using a digital caliper in the present study. Therefore, the difference between canal diameters did not influence the PBS values, which were precisely calculated for each specimen.

According to the results of the present study, due to the easy handling properties of SE resin cement and higher PBS of SE resin cement compared to other types of resin cements, SE resin cements seem to be an appropriate option for fiber post cementation in severely destroyed anterior primary teeth.

However, this study had some limitations that should be acknowledged. First, it is essential to note that this study was conducted in vitro, which limits the direct translation of the findings to clinical settings. Therefore, additional clinical studies should be conducted to assess the effect of different types of resin cements on the clinical longevity of restorations using fiber posts as an intracanal retention method in primary anterior teeth.

Furthermore, no root canal irrigants were used before fiber post cementation in this study. In future studies, it would be valuable to investigate the influence of root canal irrigants combined with different types of resin cements on the PBS of fiber posts in primary teeth. Moreover, the bond durability of different types of resin cements used for cementing fiber posts in primary teeth should be assessed in future studies.

Additionally, the effects of various radicular dentin pretreatments, such as the application of nanoparticles for dentin pretreatments, and different root canal filling materials, on the bond strength of resin cements to root canal dentin should be explored in future studies [40, 41]. The impact of silane application on the bond strength of fiber posts to resin cements warrants further investigation.

Lastly, future studies should consider evaluating the influences of complex forces, temperature changes, and functional forces in the oral environment, as these factors can affect the long-term performance of fiber posts and resin cements. Incorporating these aspects would provide a more comprehensive understanding of the clinical implications and potential challenges associated with using fiber posts and resin cements for dental restorations.

## 5. Conclusion

Based on the results of this study, using the SE resin cement for fiber post cementation in primary anterior teeth showed the best results, followed by the E&R resin cement. The lowest bond strength was observed for GIC, followed by the SA resin cement.

## Figures and Tables

**Table 1:** The manufacturer and chemical composition of the materials utilized for cementing fiber posts in the current study.

Material	Type	Manufacturer	Composition
GC GOLD LABEL 1	Glass ionomer cement	GC Corporation, Tokyo, Japan	Powder: fluoro glass alumino-silicate (amorphous). Liquid: distilled water, polyacrylic acid, 2-HEMA, UDMA

Filtek Ultimate Flowable	Nanofill flowable composite resin	3M ESPE, St. Paul, MN, USA	The resin matrix: Bis-GMA, UDMA, Bis-PMA, TEGDMA, procrylat resins The filler: 75 nm silica nanofiller 15–20 nm zirconia nanofiller ytterbium trifluoride filler (filler content: 65 wt%–46 vol %)

Variolink II	Etch-and-rinse resin cement	Ivoclar Vivadent, Schaan, Liechtenstein	Bis-GMA, UDMA, TEGDMA, barium glass, ytterbium trifluoride, Ba–Al-fluorosilicate glass, zirconia/silica, benzoyl peroxide, initiators, stabilizers, and pigments (filler content: 71 wt %)

Panavia F2.0	Self-etch resin cement	Kuraray Medical Inc., Tokyo, Japan	ED Primer II (liquid A + B): pH 2.4 Liquid A: 10-MDP, 5-NMSA, HEMA, accelerators, water Liquid B: 5-NMSA, accelerators, catalysts, water Paste-A: 10-MDP, hydrophobic aromatic dimethacrylate, hydrophobic aliphatic dimethacrylate, hydrophilic aliphatic dimethacrylate, silanated silica filler, silanated colloidal silica, dl-camphorquinone, catalysts, initiators Paste-B: Hydrophobic aromatic dimethacrylate, hydrophobic aliphatic dimethacrylate, hydrophilic aliphatic dimethacrylate, silanated barium glass filler, surface-treated sodium fluoride (Filler content: 78 wt %)

TheraCem	Self-adhesive resin cement	Bisco Dental Products, Schaumburg, IL, USA	Base: calcium base filler, glass filler, Bis-GMA, fluoride components, amorphous silica, and initiators Catalyst: 10-MDP, glass fillers (filler content: 60–65wt%)

TEGDMA, triethylenglycol dimethacrylate; Bis-PMA, Bis((methacryloxypropoxy)phenyl) propane; Bis-GMA, bisphenol-A-diglycidyl methacrylate, UDMA, urethane dimethacrylate; 10-MDP, 10-methacryloyloxydecyl dihydrogen phosphate.

## Data Availability

The data that support the findings of this study are available on request from the corresponding author.
